# Stroke Risk in Patients with Gout: A Nationwide Retrospective Cohort Study in Taiwan

**DOI:** 10.3390/jcm11133779

**Published:** 2022-06-29

**Authors:** Ping-Han Tsai, Chang-Fu Kuo, Lai-Chu See, Pei-Ru Li, Jung-Sheng Chen, Wen-Yi Tseng

**Affiliations:** 1 Division of Rheumatology, Allergy and Immunology, New Taipei Municipal TuCheng Hospital (Built and Operated by Chang Gung Medical Foundation), New Taipei City 236, Taiwan; s001033@gmail.com; 2 Division of Rheumatology, Allergy and Immunology, Chang Gung Memorial Hospital, Taoyuan City 333, Taiwan; zandis@gmail.com (C.-F.K.); lichu@mail.cgu.edu.tw (L.-C.S.); 3 Division of Rheumatology, Orthopaedics and Dermatology, School of Medicine, University of Nottingham, Nottingham NG7 2RD, UK; 4 Department of Medicine, College of Medicine, Chang Gung University, Taoyuan City 333, Taiwan; 5 Department of Public Health, Chang Gung University, Taoyuan City 333, Taiwan; peili0506@cgmh.org.tw; 6 Biostatistics Core Laboratory, Molecular Medicine Research Center, Chang Gung University, Taoyuan City 333, Taiwan; 7 Center for Artificial Intelligence in Medicine, Chang Gung Memorial Hospital, Taoyuan City 333, Taiwan; rschen0404@gmail.com; 8 Division of Rheumatology, Allergy and Immunology, Chang Gung Memorial Hospital-Keelung, No. 222, Mijin Road, Keelung City 204, Taiwan

**Keywords:** gout, stroke, risk, epidemiology

## Abstract

Objectives: To estimate stroke risk in Taiwanese patients with gout. Methods: We enrolled patients from the Taiwan National Health Insurance Database, with gout diagnosed from 2000 to 2008, and followed them up until December 2018. This cohort was propensity score-matched according to birth year, sex, the date of diagnosis of gout, comorbidities, and co-medications with individuals without gout (controls) (*n* = 310,820 in each group). Stroke was defined as the primary diagnosis at discharge after the index date. To evaluate ischemic and hemorrhagic stroke risks, we calculated their incidence, hazard ratio (HR), and two-year moving average incidence rate. Results: The incidence (95% CI) and HR of ischemic stroke were lower in the gout group than in the control group in the first 3 years (incidence: 4.74 [4.60–4.88] vs. 5.17 [5.03–5.32] per 1000 person-years; HR: 0.92 [0.88–0.96]), then became significantly higher than in the control group after 3 years (incidence: 4.10 [4.04–4.16] vs. 3.81 [3.75–3.87] per 1000 person-years; HR: 1.08 [1.05–1.10]). Similarly, the incidence (95% CI) and HR of hemorrhagic stroke was lower in the gout group than in the control group in the first 3 years (incidence: 1.51 [1.43–1.59] vs. 1.70 [1.62–1.79] per 1000 person-years; HR: 0.88 [0.82–0.92]), then became significantly higher than in controls after 3 years (incidence: 1.43 [1.39–1.46] vs. 1.26 [1.22–1.29] per 1000 person-years; HR: 1.14 [1.10–1.18]). Conclusions: In Taiwan, patients with gout had higher risks of ischemic and hemorrhagic stroke after 3 years.

## 1. Introduction

Gout, the most common form of inflammatory arthritis, is characterized by monosodium urate (MSU) crystal deposition–related manifestations. Gout diagnosis depends on clinical symptoms, laboratory testing, and imaging results [1]. The typical gout symptoms include acute swelling, pain, or tenderness in one or more peripheral joints or bursae. However, it is also associated with systemic comorbidities, such as metabolic syndromes (e.g., obesity, hypertension, diabetes mellitus [DM], and dyslipidemia), cardiovascular disease, consequences of excessive alcohol intake, and chronic kidney disease [2,3,4,5,6,7].

Stroke—defined as a neurological deficit that persists for more than 24 h resulting from a vascular cause, including cerebral infarction, intracerebral hemorrhage, and subarachnoid hemorrhage (SAH)—is a significant cause of disability and death worldwide [8]. Moreover, gout is associated with vascular diseases due to endothelial dysfunction, platelet adhesiveness, and plasma thromboembolic activity [9,10]. An animal study found hyperuricemia associated with high systemic blood pressure and COX-2–mediated, thromboxane-induced vascular disease [11]. Hyperuricemia can trigger the activation of neutrophils and the production of immune mediators associated with pro-inflammatory functions. Soluble uric acid can mediate radical generation and function as a pro-oxidant [12]. These vascular inflammatory mechanisms may contribute to vascular disease development in patients with gout. Moreover, previous studies demonstrated the importance of inflammatory pathways in the pathogenesis of ischemic stroke [13,14]. Thus, gout may potentially be one of the risk factors for stroke.

However, studies on the hyperuricemia–gout–stroke relationships have reported conflicting results. Some studies have indicated that hyperuricemia and gout increase the risks of ischemic stroke, cardiovascular disease, and ischemic stroke-related mortality [15,16,17,18,19,20,21,22,23,24,25]. In contrast, others have suggested that hyperuricemia and gout are not associated with the risks of stroke or cardiovascular disease [26,27]. Furthermore, limited studies report the relationship between gout and hemorrhagic stroke, which warrants further investigation. In the present study, we estimated the ischemic and hemorrhagic stroke risks in patients with gout compared with the matched non-gout population using the Taiwan National Health Insurance (NHI) Database.

## 2. Methods

Ethical approval was obtained from the Institutional Review Board (IRB) of Chang Gung Medical Foundation (IRB number: 201800242B1). Because we analyzed anonymized data, patient consent was not required.

### 2.1. Data Source

Taiwan’s National Health Insurance was established in 1996 and covers almost the entire population of Taiwan. The NHI database records the sex, date of birth, residence, insurance details, family relationships, dates of inpatient and outpatient visits, medical diagnoses, medical expenditures, prescription details, vaccination status, examinations, operations, procedures, and fees incurred for all NHI beneficiaries. A unique personal identifier—encrypted before data are released to researchers upon request—is used to link, internally and externally, all the NHI database data with those from other databases. Consequently, all NHI beneficiary data, including those for major diseases (e.g., gout and stroke), are available to researchers.

### 2.2. Study Design and Study Population

**Figure 1:**
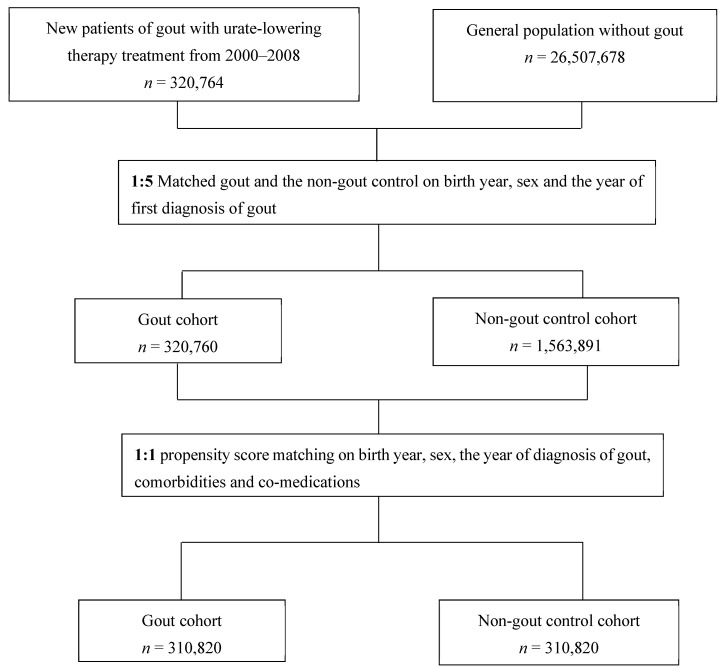
Patient selection flow.

We identified a cohort of incident patients diagnosed with gout from 2000 to 2008 in Taiwan from the NHI database (the gout cohort) and followed them up until the end of 2018. Patients with gout received a primary diagnosis of gout (International Classification of Diseases, Ninth Revision, Clinical Modification [ICD-9-CM] code: 274). To avoid misclassification (e.g., cellulitis or monoarthritis for gout), patients had to have at least two outpatient visits for gout diagnosis and were prescribed urate-lowering agents (e.g., allopurinol, febuxostat, benzbromarone, probenecid, or sulfinpyrazone). The non-gout control group were patients without diagnosis of gout (ICD-9-CM code: 274) in principal, additional, or other diagnoses from 2000 to 2008. In order to have the two compatible cohorts, the non-gout cohort was first 5:1 matched according to birth year, sex, and the year of diagnosis of gout, because of the substantial number (*n* = 26,507,678) of beneficiaries in the NHI during this study period. Next, the non-gout control cohort was 1:1 propensity score matched according to birth year, sex, the year of diagnosis of gout, comorbidities, and co-medications within a caliper width equal to 0.2, the SD of the logit of the estimated propensity score [28]. The flow of patient selection is illustrated in Figure 1.

### 2.3. Ascertainment of Outcome, Comorbidities, and Co-Medications

The incidence of ischemic and hemorrhagic stroke was calculated separately for both cohorts. We identified stroke type according to the primary diagnosis at discharge as ischemic stroke (ICD-9-CM codes during 2000–2015: 433, 434, 436; ICD-10-CM codes since 2016: I63, I64) or hemorrhagic stroke (ICD-9-CM codes during 2000–2015: 430–432, 852, 853; ICD-10-CM codes since 2016: I60–I62). The definition of stroke necessitates a hospitalization with discharge as ischemic or hemorrhagic stroke to eliminate misclassification (e.g., focal neurological sign, TIA for stroke). The accuracy of ischemic and hemorrhagic stroke diagnosis in the NHI database has been validated previously; 97.85% of an NHI database sample was confirmed through radiology examination and clinical presentation [29]. 

Baseline covariates were obtained from claim records with the diagnoses, medications, or procedures codes before the index date. A history of any prescription medicine was confined to medications taken at least once within 3 months before the index date. The definition and coding of comorbidities are shown in Supplemental Table S1.

### 2.4. Statistical Analysis

The propensity score was obtained using the generalized boosted model (GBM), which included demographic variables, comorbidities, and co-medications (Table 1). The advantages of GBM automatically determine the best functions of covariates to achieve an optimal balance between the two cohort groups, and it is less affected by extreme weights [30]. The absolute standardized mean difference (ASMD) was used to measure the balance of demographic variables, comorbidities, and co-medications between the two cohorts. An ASMD larger than 0.1 was considered statistically significant [31].

The two groups were followed from the index date until a stroke, death, or 31 December 2018, whichever occurred first. The incidence is the number of event occurrences divided by the total following person-year. A 2-year moving average of the stroke incidence rate was calculated to determine how the rate varied with time after the diagnosis of gout (index date). The 95% confidence interval (CI) of the incidence rate was based on the relationship between the F distribution and binomial distribution [32]. Cox’s proportional hazards model was used to obtain the hazard ratio (HR) with 95% (CIs) for stroke in the gout cohort, with the non-gout cohort as a reference group. Note that only the gout status vs. non-gout status was included in the Cox model because the two cohorts were compatible in demographics, comorbidities, and co-medications [33]. All statistical analyses were performed using SAS (version 9.4; SAS Institute, Cary, NC, USA).

## 3. Results

Table 1 presents the demographic and clinical characteristics of the gout and control cohorts. In both cohorts, the proportion of men was around 80%, and the average age was approximately 50. Most people lived in rural areas, with moderate income (quartile 3). The most common co-medications in both cohorts were nonsteroidal anti-inflammatory drugs (NSAIDs), H2-blockers, angiotensin-converting enzyme inhibitors (ACEI), angiotensin II receptor antagonists (ARB), and antiplatelet agents. The most common comorbidities were hypertension, diabetes mellitus, dyslipidemia, and chronic liver disease in both cohorts. About 1% of patients had ischemic stroke or hemorrhagic stroke.

Table 2 compares the two cohorts’ ischemic and hemorrhagic stroke incidences and HRs. The incidence (95% CI) of ischemic stroke was significantly higher in the patients with gout than in the controls (5.06 [5.00–5.13] vs. 4.87 [4.81–4.94] per 1000 person-years). The hazard ratio (HR) (95% CI) for ischemic stroke was 1.04 (1.02–1.06). However, the incidence (95% CI) and HR of ischemic stroke were lower in the gout group than in the control group in the first 3 years (incidence: 4.74 [4.60–4.88] vs. 5.17 [5.03–5.32] per 1000 person-years; HR: 0.92 [0.88–0.96]), then became significantly higher than in the controls after 3 years (Incidence: 4.10 [4.04–4.16] vs. 3.81 [3.75–3.87] per 1000 person-years; HR: 1.08 [1.05–1.10]).

Compared with the controls, the patients with gout also had a higher incidence (95% CI) of hemorrhagic stroke (1.73 [1.69–1.77] vs. 1.60 [1.56–1.64] per 1000 person-years), and the HR (95% CI) for hemorrhagic stroke was 1.08 (1.05–1.12). However, the incidence (95% CI) and HR of hemorrhagic stroke were lower in the gout group than in the control group in the first 3 years (incidence: 1.51 [1.43–1.59] vs. 1.70 [1.62–1.79] per 1000 person-years; HR: 0.88 [0.82–0.92]), then became significantly higher than in controls after 3 years (incidence: 1.43 [1.39–1.46] vs. 1.26 [1.22–1.29] per 1000 person-years; HR: 1.14 [1.10–1.18]).

**Figure 2:**
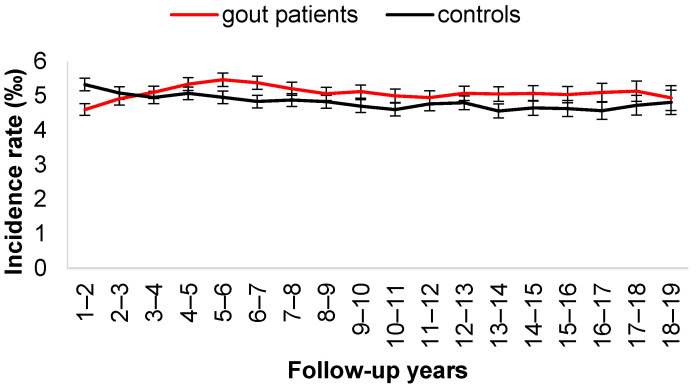
Two-year moving average of incident rate of ischemic stroke.

**Figure 3:**
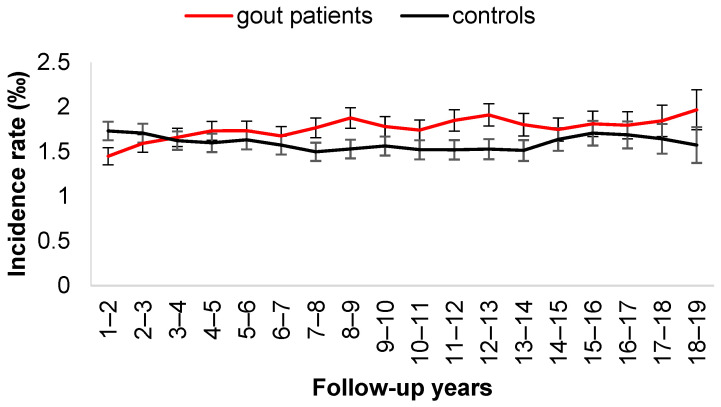
Two-year moving average of incident rate of hemorrhagic stroke.

Figure 2 depicts the two-year moving average incidence rate of ischemic stroke. The incidence rate of ischemic stroke was higher in the control cohort in the first 3 years and became significantly higher in the gout cohort after 3 years. Figure 3 shows the two-year moving average incidence rate of hemorrhagic stroke. Similarly, the incidence rate of hemorrhagic stroke was higher in the control cohort in the first 3 years and then became higher in the gout cohort after 3 years.

## 4. Discussion

For this nationwide population, we analyzed the relationship between gout and stroke. The ischemic stroke incidence was threefold higher in patients with gout than the incidence of hemorrhagic stroke. More importantly, we saw that the HR of ischemic stroke was lower in the gout group than in the control group in the first 3 years and then became significantly higher than in the controls after 3 years. A similar phenomenon was seen for hemorrhagic stroke. 

The relationship between hyperuricemia, gout, and overall stroke (including ischemic and hemorrhagic stroke) has been discussed previously. Regarding overall stroke risk, Holme et al. [19] measured serum uric acid levels in 417,734 men and women during health check-ups in Sweden and found that the higher the serum uric acid levels, the higher the overall stroke risks (HR [95% CI] for overall stroke: 1.09 [1.07–1.11] in men and 1.13 [1.10–1.15] in women). Because gout is associated with MSU crystallization, which triggers downstream inflammatory responses [34,35], inflammation may also contribute to stroke [36]. Thus, investigating the gout–stroke relationship, rather than the hyperuricemia–stroke relationship, is essential. In the United Kingdom, Seminog et al. used a national dataset of hospital admissions and death records from 1999 to 2011 and found an increased overall stroke risk in patients with gout (risk ratio (RR) [95% CI]: 1.71 [1.68–1.75]) [16].

Moreover, Singh et al. used US claims data from 2007 to 2010 and reported that gout incurred an overall incident stroke risk equivalent to that of DM, indirectly demonstrating the relationship of gout with overall stroke [15]. The results of these database-based studies demonstrate that gout increases overall stroke risk. However, these results contradict those of Clarson et al., who used the data from the United Kingdom Clinical Practice Research Datalink and found HRs (95% CIs) for the overall stroke of only 0.93 (0.81–1.06) in men and 1.34 (1.15–1.57) in women [26]. However, Clarson et al. included only older patients from the United Kingdom, enrolled from 1987 to 1999, whereas the present cohort comprised a younger population from Taiwan, enrolled from 2000 to 2008. The differences can be explained further by the low prevalence of gout in older adults in earlier studies and by the high prevalence of gout in Asian-Pacific countries [37].

Among all relevant studies, the present study included the largest patient population. Furthermore, we classified patients to have overall stroke and gout only when they were hospitalized for stroke and were prescribed urate-lowering agents, respectively. Thus, the use of the relatively strict definition for the inclusion of gout cases may have contributed to the contrast between current results and the previous findings.

Ischemic stroke occurs when a blood vessel supplying blood to the brain is obstructed, whereas hemorrhagic stroke occurs when a weakened blood vessel ruptures. Consequently, these two stroke types have different outcomes [38], necessitating separate calculations of their risks. However, data on ischemic and hemorrhagic stroke risks calculated separately are limited. Holme et al. reported that elevated serum uric acid levels increased ischemic and hemorrhagic stroke risks—with HRs (95% CIs) of 1.10 (1.07–1.12) and 1.07 (1.01–1.12) in men, respectively, and 1.15 (1.12–1.17) and 1.08 (1.02–1.15) in women, respectively [19]. Moreover, Seminog et al. reported that patients with gout had increased ischemic and hemorrhagic stroke risks—with RRs (95% CIs) of 1.68 (1.64–1.73) and 1.69 (1.61–1.77), respectively [16]. Another study demonstrated that hyperuricemia might increase arterial stiffness and inflammation, supporting our finding that gout increases stroke risk [39]. In this study, the gout group has a lower HR of ischemic and hemorrhagic stroke than the control group in the first 3 years and the other way around afterward. The reason for lower ischemic and hemorrhagic stroke incidence in the gout cohort in the first 3 years was unclear. Because the definition of gout in our study requires the use of a urate-lowering agent at enrolment, a previous study showed urate-lowering agents might reduce the incidence of stroke in gout patients [40]. Thus, the temporal lower incidence of stroke in the first 3 years may be because of the interference of urate-lowering agents. Moreover, strict diet control and better drug compliance may be observed in the first 3 years after the diagnosis of gout. Therefore, the loss of the protective effect of the urate-lowering agent after the first 3 years may be related to poor adherence as time passes. Further prospective or randomized studies are suggested to investigate this temporal relationship.

We previously reported that the comorbidities of gout include cardiovascular diseases, renal diseases, the consequences of total joint replacement, and cancer [41,42,43,44,45,46]. In this study, the stroke risk was high in the gout population from Taiwan, indicating the need of monitoring gout comorbidities and stroke incidents in clinical practice. Moreover, gout can induce multiple comorbidities, influencing treatment outcome and survival [2,3,4,5,6,7]. Therefore, the management of gout should include the treatment of not only arthritis, but also systemic diseases. Consequently, considering the use of a comorbidity checklist when treating patients newly diagnosed with gout and those with existing gout is crucial.

However, this study had several drawbacks that limited the generalization of our results. First, we did not adjust the data for body mass index, smoking, dietary factors, and alcohol consumption because these data were unavailable in the NHI database. Second, we could not include serum uric acid level data or tophus formation. However, a high serum uric acid level and tophus formation reflect severe gouty arthritis, which may increase stroke risk. Third, we could not investigate whether lowering serum uric acid levels by taking urate-lowering agents reduces stroke risk, and we did not identify the urate-lowering agents that may be the most effective in this context. Further relevant exploration is thus warranted. Fourth, this study’s incidence of ischemic and hemorrhagic stroke required hospitalization. In other words, the incidence rates of the two strokes did not cover those who sought treatment through the emergency department, but were not admitted to the hospital. Fifth, the propensity score matching between the gout and non-gout cohorts allows a good balance of comorbidity and medication use at the index date, but the comorbidity and medication use might change over time, which is particularly serious in our study with a long follow-up time. Perhaps, the nested case-control study might sort out this problem [47]. 

In conclusion, in this population-based study in Taiwan, patients with gout demonstrated an increased stroke risk, for both ischemic and hemorrhagic stroke. Moreover, ischemic stroke incidence was threefold higher in these patients with gout than the incidence of hemorrhagic stroke. Therefore, in general, physicians should be aware of the risk of stroke in gout patients and apply relevant prevention measures.

## Figures and Tables

**Table 1:** Demographic and clinical characteristics of gout and non-gout control cohorts in Taiwan, 2000 to 2008.

	Gout (*n* = 310,820)	Controls (*n* = 310,820)	ASMD
Male, No. (%)	248,136 (79.83)	248,039 (79.80)	0.0008
Age, mean (SD), y	50.93 (16.80)	52.55 (16.68)	0.0963
Age group, No. (%)			0.1863
<10	36 (0.01)	34 (0.01)	
10–19	7286 (2.34)	6806 (2.19)	
20–29	27,392 (8.81)	22,449 (7.22)	
30–39	48,302 (15.54)	43,097 (13.87)	
40–49	68,875 (22.16)	65,792 (21.17)	
50–59	55,445 (17.84)	57,498 (18.50)	
60–69	51,846 (16.68)	56,369 (18.14)	
70–79	40,888 (13.15)	47,044 (15.14)	
80–89	10,043 (3.23)	11,061 (3.56)	
≥90	707 (0.23)	670 (0.22)	
Place of residence, No. (%)			0.0000
Urban	81,614 (26.26)	81,635 (26.26)	
Suburban	81,490 (26.22)	81,217 (26.13)	
Rural	137,283 (44.17)	137,319 (44.18)	
Unknown	10,433 (3.36)	10,649 (3.43)	
Monthly income levels, No. (%)			0.0000
Quartile 1 (=dependent)	61,715 (19.86)	62,547 (20.12)	
Quartile 2 (<NTD 15,000)	44,897 (14.44)	44,762 (14.40)	
Quartile 3 (<NTD 25,000)	137,296 (44.17)	137,033 (44.09)	
Quartile 4 (≥NTD 25,000)	66,912 (21.53)	66,478 (21.39)	
Occupation, No. (%)			0.0000
Civil servants, teachers, military personnel, and veterans	32,895 (10.58)	33,026 (21.78)	
Private business workers	101,714 (32.72)	101,169 (32.55)	
Union organization workers	123,892 (39.86)	124,341 (40.00)	
Other	52,319 (16.83)	52,284 (16.82)	
Medication use			
NSAIDs	27,849 (8.96)	27,951 (8.99)	0.0011
PPI	2968 (0.95)	3116 (1.00)	0.0048
H2 blocker	36,505 (11.74)	37,400 (12.03)	0.0089
ACEI and ARB	24,227 (7.79)	24,582 (7.91)	0.0042
*β*-blocker	38,128 (12.27)	39,112 (12.58)	0.0096
Verapamil or Diltiazem	8275 (2.66)	8632 (2.78)	0.0071
Statin	7988 (2.57)	7892 (2.54)	0.002
Antiplatelet agent	19,530 (6.28)	20,223 (6.51)	0.0091
Oral anticoagulant	1071 (0.34)	1069 (0.34)	0.0001
Systemic diseases			
Hypertension	21,274 (6.84)	20,671 (6.65)	0.0077
Diabetes mellitus	18,005 (5.79)	18,512 (5.96)	0.0069
Dyslipidemia	19,651 (6.32)	19,274 (6.20)	0.005
Chronic liver disease	11,099 (3.57)	10,611 (3.41)	0.0086
Chronic kidney disease	4054 (1.30)	3666 (1.18)	0.0113
Chronic lung disease	2762 (0.89)	2787 (0.90)	0.0009
Congestive heart failure	504 (0.16)	495 (0.16)	0.0007
Chronic ischemic heart disease	2354 (0.76)	2077 (0.67)	0.0106
Ischemic stroke	1947 (0.63)	2761 (0.89)	0.0302
Hemorrhagic stroke	598 (0.19)	818 (0.26)	0.0148
Atrial fibrillation	2770 (0.89)	2913 (0.94)	0.0048
PCI	947 (0.30)	840 (0.27)	0.0064
CABG	275 (0.09)	229 (0.07)	0.0052
Cancer	4023 (1.29)	4064 (1.31)	0.0012

ACEI: angiotensin-converting enzyme inhibitor; ARB: angiotensin II receptor antagonists; ASMD: absolute standardized mean difference; CABG: coronary artery bypass grafting; NSAIDs: nonsteroidal anti-inflammatory drugs; PCI: percutaneous coronary intervention; PPI, proton pump inhibitor.

**Table 2:** Incidence, hazard ratios, and follow-up year for ischemic and hemorrhagic stroke among gout and non-gout cohorts in Taiwan, 2000 to 2008.

	Gout	Controls
**Ischemic Stroke**		
Follow-up year (mean ± SD)	13.98 ± 4.96	13.98 ± 4.96
Incidence/1000 person-years (95% CI)	5.06 (5.00–5.13)	4.87 (4.81–4.94)
No. of case, (%)	22,179 (7.14)	20,983 (6.75)
Hazard Ratio (95% CI) [*p* value]	1.04 (1.02–1.06) [<0.0001]	reference
1–3 years		
Incidence/1000 person-years (95% CI)	4.74 (4.60–4.88)	5.17 (5.03–5.32)
No. of case, (%)	4329 (1.39)	4676 (1.50)
Hazard Ratio (95% CI) [*p* value]	0.92 (0.88–0.96) [<0.0001]	reference
4+		
Incidence/1000 person-years (95% CI)	4.10 (4.04–4.16)	3.81 (3.75–3.87)
No. of case, (%)	17,850 (6.01)	16,307 (5.58)
Hazard Ratio (95% CI) [*p* value]	1.08 (1.05–1.10) [<0.0001]	reference
**Hemorrhagic stroke**		
Follow-up year (mean ± SD)	14.24 ± 4.75	14.24 ± 4.75
Incidence/1000 person-years (95% CI)	1.73 (1.69–1.77)	1.60 (1.56–1.64)
No. of case, (%)	7723 (2.48)	7022 (2.26)
Hazard Ratio (95% CI) [*p* value]	1.08 (1.05–1.12) [<0.0001]	reference
1–3 years		
Incidence/1000 person-years (95% CI)	1.51 (1.43–1.59)	1.70 (1.62–1.79)
No. of case, (%)	1382 (0.44)	1547 (4.98)
Hazard Ratio (95% CI) [*p* value]	0.88 (0.82–0.92) [0.0009]	reference
4+		
Incidence/1000 person-years (95% CI)	1.43 (1.39–1.46)	1.26 (1.22–1.29)
No. of case, (%)	6341 (2.12)	5475 (1.86)
Hazard Ratio (95% CI) [*p* value]	1.14 (1.10–1.18) [<0.0001]	reference

## Data Availability

Not applicable.

## Supplementary Materials

The following are available online at https://www.mdpi.com/article/10.3390/jcm11133779/s1, Table S1: International Classification of Disease, Ninth Revision, Code or Procedure Codes for Comorbidities.
