# Supplementary material for: Stroke Risk in Patients with Gout: A Nationwide Retrospective Cohort Study in Taiwan
Source: J Clin Med. 2022 Jun 29;11(13):3779. doi: 10.3390/jcm11133779 (PMC9267343; doi:10.3390/jcm11133779)
Supplement: Supplementary file 1 [file jcm-11-03779-s001.zip › jcm-1765045-supplementary.pdf]

Table S1. International Classification of Disease 9th revision code or procedure codes for comorbidities.

Disease	Coding	Requirement
Hypertension	401-402	Outpatient department ≥2
Diabetes mellitus	250	Outpatient department ≥2
Dyslipidemia	272	Outpatient department ≥2
Chronic liver disease	570, 571, 572	Outpatient department ≥2
Chronic kidney disease	580, 581, 582, 583, 584, 585, 586, 587, 588, 589	Outpatient department ≥2
Chronic lung disease	490, 491.0, 491.1, 491.20-491.22, 491.8, 491.9, 492.0, 492.8, 493.00-493.02 493.10-493.12, 493.20-493.22, 493.81, 493.82, 493.90-493.92, 494.0, 494.1, 495.8, 495.9, 496, 500, 502, 503, 504, 505	Primary discharge diagnosis
Congestive heart failure	428	Primary discharge diagnosis
Chronic ischemic heart disease	410, 411, 412, 413, 414	Primary discharge diagnosis
Percutaneous coronary intervention	NHI procedure code: 33076A, 33077A, 33078A, 33076B, 33077B, 33078B	Discharge
Coronary artery bypass graft	NHI procedure code: 68023A, 68023B, 68024A, 68024B, 68025A, 68025B	Discharge
Cancer	140-208	Outpatient department ≥2
